# Detection and Outcome of Endocervical Atypia in Cytology in Primary HPV Screening Programme

**DOI:** 10.3390/diagnostics11122402

**Published:** 2021-12-20

**Authors:** Johanna Pulkkinen, Saara Kares, Heini Huhtala, Ivana Kholová

**Affiliations:** 1 Pathology, Fimlab Laboratories, Arvo Ylpön katu 4, 33520 Tampere, Finland; johanna.pulkkinen@fimlab.fi (J.P.); saara.kares@fimlab.fi (S.K.); 2 Faculty of Social Sciences, Tampere University, Arvo Ylpön katu 34, 33520 Tampere, Finland; heini.huhtala@tuni.fi; 3 Faculty of Medicine and Health Technology, Tampere University, Arvo Ylpön katu 34, 33520 Tampere, Finland

**Keywords:** HPV, screening, cytology, endocervical adenocarcinoma, adenocarcinoma in situ, atypical endocervical cells

## Abstract

Most endocervical adenocarcinomas (EAC) are associated with high-risk HPV (hrHPV) infection, with HPV genotypes 16, 18 and 45 accounting for >90% of the cases. Among endocervical glandular lesions, screening with hrHPV test has previously shown to predict the outcome better than cytology, although around one-fifth of the EAC remain negative both in hrHPV testing and cytology. The study consists of two consecutive HPV-primary screening rounds, conducted in 2012–2015 and 2017–2020. Of the 87 women aged 35 to 60 years of age diagnosed with Atypical endocervical cells, NOS or Atypical endocervical cells, favor neoplastic cytology during the first screening round, 63 (72.4%) were hrHPV positive and 24 (27.6%) were hrHPV negative. Among hrHPV positive patients, three EAC, two adenocarcinomas in situ (AIS), one AIS + high-grade intraepithelial lesion (HSIL) and 13 HSIL were found. Of the histologically verified lesions, 68.4% (13/19) were purely of squamous origin. All the EAC and AIS were HPV16 or HPV 18 positive. No high-grade histological lesions were found among the hrHPV negative patients with cytological glandular atypia. A later database search revealed one HPV-negative, gastric-type mucinous EAC that was missed by the HPV primary screening.

## 1. Introduction

In Finland, national cervical cancer screening has been organized since the 1960s. Women from the age of 30 to the age of 60 are invited to participate every fifth year, and in some municipalities 25- and 65-year-olds are also included. Since the beginning of the organized screening, the number of cervical cancer deaths in Finland has decreased to one-fifth of its original number [1]. Nowadays, Finland is among the European countries with the lowest rates of cervical cancer incidence [2].

Traditionally, the conventional Pap smear has been the primary test. In 2012, the city of Tampere, and later all municipalities in Pirkanmaa region, started HPV primary screening with high-risk HPV (hrHPV) test and cytological smear as a triage in women aged ≥35 years [3,4,5,6].

The important role of hrHPV infection leading to the development of invasive cervical squamous cell carcinoma (SCC) and behind the most of the endocervical adenocarcinomas (EAC) has been well documented [7,8,9,10]. In the previous studies, based on DNA detection of whole-tissue sections, 62% to 75% of the EAC were reported to be HPV positive with HPV genotypes 16, 18 and 45 accounting for 90% to 94.1% of the positive cases [8,9,10]. Of the EAC subtypes, the usual subtype is the most common, with the reported relative portion of 59% to 74.6% of all EAC [9,10]. The usual subtype also presents with the strongest association with HPV infection, with 60% to 82% of the tumors found to be positive [9,10].

In the previous studies, the clear cell adenocarcinomas (AC) accounted for 3.9% to 4%, the serous AC for 3% to 3.2%, the endometrioid AC for 1.4% to 3%, the minimal deviation AC 1.6% to 6% and the AC, not otherwise specified, for 4.7% to 8% of the EAC [9,10]. These less common EAC subtypes showed significantly lower prevalence for hrHPV, with only 13% to 20% of the clear cell AC, 0% to 25% of the serous AC, 13% to 27.3% of the endometrioid AC, 0% to 8.3% of the minimal deviation AC and 13.9% to 24% of the AC, not otherwise specified as positive [9,10].

In previous studies, HPV testing identified earlier more women with cancer or precancerous lesions than cytology alone [11,12,13,14,15,16]. Especially among endocervical glandular lesions, the HPV test predicted the outcome better than the cytology. Of the EAC 78% and 79.0% and of the adenocarcinoma in situ (AIS) cases 80% and 82.2% were detected by positive HPV screening test in comparison to detection of only 15% and 45.4% of EAC and 40% and 53.2% of AIS by cytology alone [11,12]. A negative HPV test result also predicted a negative end result better than a negative cytological sample alone [11,12,13]. Yet, 22% of the EAC and around 15% of the AIS have been reported to remain negative both in hrHPV testing and cytology [11,12].

The aims of the present study are to evaluate the detection and outcome of endocervical atypia in an HPV primary screening programme.

## 2. Materials and Methods

The study cohort represents women participating in an HPV primary screening programme organized by Fimlab Laboratories during 2012–2015 in the Pirkanmaa region, Finland. During these years, altogether 93.439 women aged 35 to 60 years were invited to cervical cancer screening, which included both the hrHPV test and the conventional pap test. All the pap tests of the hrHPV positive women were analyzed and, as a quality assurance, 10% of the HPV negative patients were assessed cytologically in the 2012–2016 period.

In total, 66.147 (70.8%) of the invited women participated in the screening. Out of the participants, 87 (0.13%) were diagnosed with endocervical glandular atypia (Atypical Endocervical Cells, NOS or Atypical Endocervical Cells, Favor Neoplastic) with or without a squamous atypia (Atypical Squamous Cells, Undetermined Significance, Low-Grade Squamous Intraepithelial Lesion, High-Grade Squamous Intraepithelial Lesion, Atypical Squamous Cells, Cannot Exclude HSIL).

At the beginning of the study, according to the screening protocol and the national guidelines, women with a positive hrHPV test result and/or a cytological diagnosis of Atypical Squamous Cells, Undetermined Significance (ASC-US) and/or Atypical Endocervical Cells, NOS (AEC, NOS) were referred to repeat sampling after 12 months [17]. The repeat test included both the hrHPV test and the conventional Pap test. If the repeated hrHPV test was found to be positive and/or there was a cytological atypia, the woman was referred for a colposcopy. If the hrHPV test was negative and there was no cytological atypia, the patient received an invitation to the next screening round in five years from the original invitation.

In 2016, there was a change in the national guidelines after which patients with cytological diagnosis of AEC, NOS with or without a positive hrHPV test result were referred for a colposcopy immediately.

Women with a cytological diagnosis of Atypical Endocervical Cells, Favor Neoplastic (AEC, FN) and/or a squamous cytological diagnosis of Low-Grade Squamous Intraepithelial Lesion (LSIL) or worse were always immediately referred for a colposcopy despite the hrHPV status of the patient. After the colposcopy, the patient was treated according to the national guidelines [17]. Despite the colposcopic findings or the procedures they lead in to, all women received an invitation to the next screening round after five years from the original invitation. 

At the first screening round in 2012–2015, the patients were between 35 to 60 years old, and at the second screening round in 2017–2020, they were between the ages of 40 to 65 years.

All diagnoses on cervical cytological samples were provided according to the Bethesda Classification for Reporting Cervical Cytology 2014 [18,19,20]. The Abbot RealTime hrHPV PCR assay (RealTime; Abbot, Wiesbaden, Germany) was used for the detection of the hrHPV DNA. The test recognizes 14 hrHPV genotypes, including types 16, 18, 31, 33, 35, 39, 45, 51, 52, 56, 58, 59, 66 and 68. The hrHPV genotypes 16 and 18 were reported separately and the rest of the genotypes were reported as “other hrHPV than 16 or 18”.

The HPV status, genotype, cytological and histological data were retrieved from the Laboratory Information System (LIS) of the Fimlab Laboratories Oy. Later, an additional LIS- search was conducted to find possible EACs missed by the primary HPV screening during the study period 2012 to 2020. 

For the statistical analysis, SPSS version 25 was used (IBM SPSS Statistics for Windows, version 25.0, IBM Corporation, Armonk, NY, USA).

Because the samples of the study include those produced by the national cervical cancer screening protocol and its follow-ups, the individual consent of each participant was not requested. The study was approved by the Ethical committee of Pirkanmaa Health Care District (R13094 and R16022). The study was conducted according to the Declaration of Helsinki.

## 3. Results

**Figure 1:**
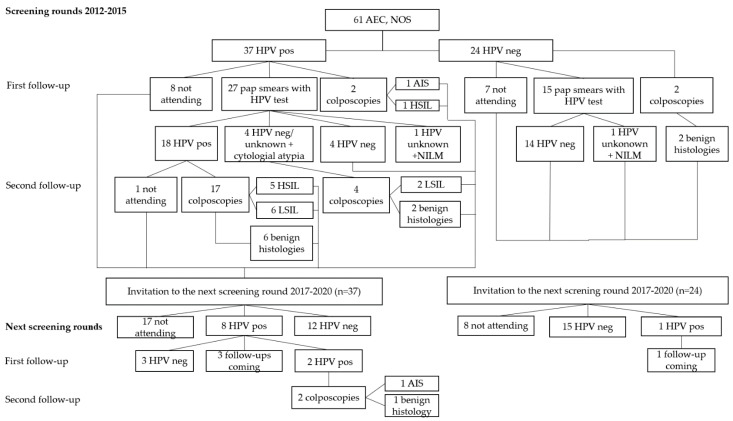
The outcome of two consecutive HPV primary cervical cancer screening rounds of patients with atypical endocervical cells, NOS in cytology. Abbreviations representing cytological diagnoses: AEC, NOS, Atypical Endocervical Cells, NOS; NILM, Negative for Intraepithelial Lesion or Malignancy. Abbreviations representing histological diagnoses: AIS, Adenocarcinoma in Situ; HSIL, High-Grade Intraepithelial Lesion; LSIL, Low-Grade Intraepithelial Lesion; Other abbreviations: HPV, human papillomavirus; pos, positive; neg, negative.

After the first screening rounds in 2012–2015, 61 patients were diagnosed as AEC, NOS on cytology (Figure 1). Of those patients, 37 were hrHPV positive and presented with one adenocarcinoma in situ (AIS) and six high-grade intraepithelial lesions (HSIL) on follow-ups in the first screening rounds. During the second screening rounds in 2017–2020, a patient with HSIL-histology on the first screening round was diagnosed with an additional AIS. The diagnosis of AIS was reached after 5 years and 9 months from the first screening sample and from the first cytological diagnosis of endocervical glandular atypia. The patient was initially positive with hrHPV genotypes 16, 18 and an hrHPV type other than 16 or 18 (Table 1). At the time of the AIS diagnosis, HPV16 persisted.

**Figure 2:**
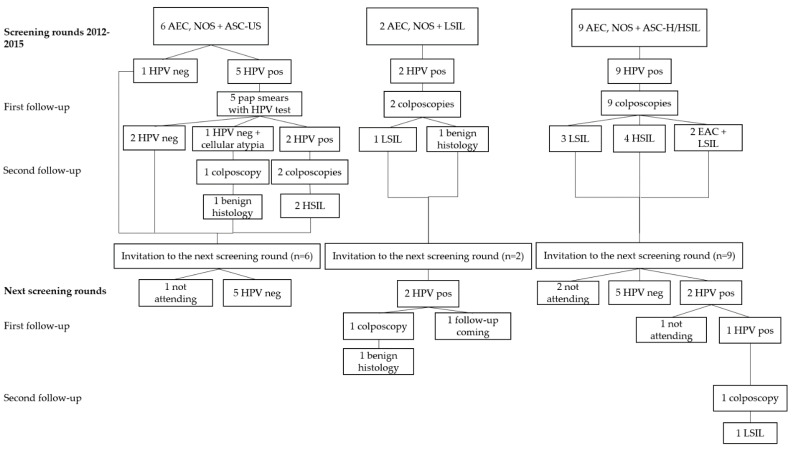
The outcome of two consecutive HPV primary cervical cancer screening rounds of patients with atypical endocervical cells, NOS and a squamous cell atypia in cytology. Abbreviations: AEC, NOS, Atypical Endocervical Cells, NOS; EAC, Endocervical Adenocarcinoma; ASC-US, Atypical Squamous Cells, Undetermined Significance; LSIL, Low-Grade Squamous Intraepithelial Lesion; HSIL, High-Grade Squamous Intraepithelial Lesion; ASC-H, Atypical Squamous Cells, Cannot Exclude an HSIL; HPV, human papillomavirus; pos, positive; neg, negative. Combinations of abbreviations representing cytological diagnoses: AEC, NOS + ASC-US; AEC, NOS + LSIL; AEC, NOS + ASC-H/HSIL. Abbreviations representing histological diagnoses: LSIL; HSIL; EAC + LSIL.

In 2012–2015 a total of 17 patients were diagnosed with AEC, NOS in combination with a squamous cytological diagnosis (Figure 2). Out of these patients, 16 were hrHPV positive. During the follow-ups, two histological HSIL were found behind the diagnoses of AEC, NOS + ASC-US. The combination of AEC, NOS + HSIL/Atypical Squamous Cells, Cannot Exclude an HSIL (ASC-H) resulted in four histological HSIL and to two combinations of EAC and LSIL. No additional high-grade histological lesions were found during the next screening rounds in 2017–2020.

**Figure 3:**
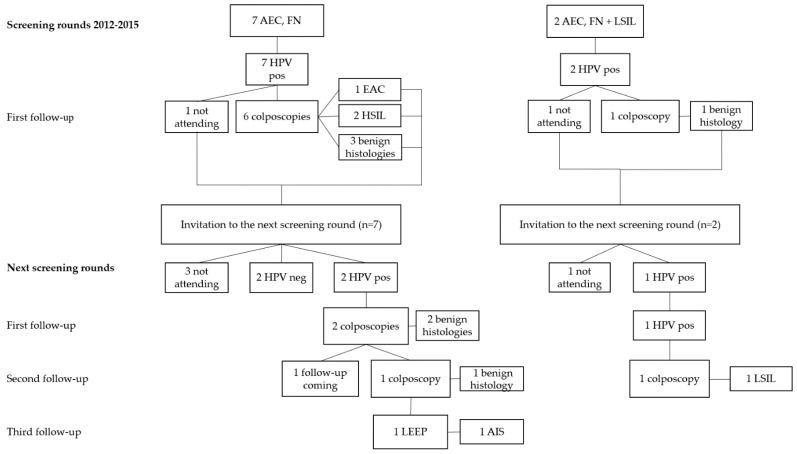
The outcome of two consecutive HPV primary cervical cancer screening rounds of patients with atypical endocervical, favoring neoplastic with or without a squamous cell atypia in cytology. Abbreviations representing cytological diagnoses: AEC, FN, Atypical Endocervical Cells, Favor Neoplastic; AEC, FN + LSIL, Low-Grade Squamous Intraepithelial Lesion. Abbreviations representing histological diagnoses: EAC, Endocervical Adenocarcinoma; LSIL, Low-Grade Squamous Intraepithelial Lesion; HSIL, High-Grade Squamous Intraepithelial Lesion. Other abbreviations: HPV, human papillomavirus; pos, positive; neg, negative; LEEP, loop electrosurgical excision procedure.

A cytological diagnosis AEC, FN was provided seven times in the first screening rounds in 2012–2015 and AEC, FN + LSIL was diagnosed two times (Figure 3). All patients were hrHPV positive. In the first group, two HSIL and one EAC was diagnosed during the follow-ups at the first screening rounds and one AIS on the follow-ups during the second screening rounds. The diagnosis of AIS was reached 6 years and 11 months after the first screening sample, with cytological suspicion of endocervical glandular neoplasia. During the time, the patient presented with persistent HPV18 infection (Table 1). In the latter group, no high-grade histological lesions were found in either the first or the second screening round.

Altogether, behind the 87 cytological endocervical glandular diagnoses with or without combined squamous cytological atypia, one EAC, two AIS, two EAC + LSIL, one AIS + HSIL and 13 HSIL were found reflecting that 68.4% (13/19) of the high-grade lesions verified histologically were purely of squamous origin (Table 1). There are still six follow-ups planned and yet to be conducted among patients initially positive with hrHPV and with a persistent infection. 

Of the endocervical malignancies, 33.3% (2/6) were HPV18 positive and 66.7% (4/6) were HPV 16 positive (Table 1). Among HSIL, 42.9% (6/14) represented hrHPV genotypes other than HPV16 or HPV18, 28.7% (4/14) HPV16 and 28.7% (4/14) a combination of HPV16 and/or HPV18 and hrHPV type other than HPV16 or HPV18. The investigated HPV types (16, 18 or hrHPV other than 16 or 18), or a combination of them, showed no statistical association (*p*-value > 0.05) to endocervical malignancies or to squamous neoplasias.

Aside for the two above-described AIS cases with the wide time lag before the histological diagnosis, all the high-grade histological lesions were diagnosed during the first screening round. No high-grade histological lesions were found among patients with cytological endocervical glandular atypia and a negative hrHPV test result (Figure 1, Figure 2 and Figure 3, Table 2). There was one HPV-negative gastric-type mucinous EAC missed by the HPV primary screening. The pap test of the patient was not analyzed, that is to say, the patient was not included in the 10% of the HPV-negative cases assessed cytologically as a quality assurance.

The diagnosis of this gastric-type mucinous EAC was made two years after the negative HPV primary screening result. The diagnosis was immunohistochemically confirmed, and the patient was treated with hysterectomy, combined with salpingo-oophorectomy and lymphadenectomy. There were no metastases. The tumor itself was not tested for hrHPV, but the vaginal hrHPV tests have remained negative since. A 5-year follow-up has been conducted, and no additional tumors were observed.

During the first screening round, 17.5% (11/63) of the hrHPV positive patients and 29.2% (7/24) of the hrHPV negative patients left follow-ups unattended (Table 2). Of the originally hrHPV-negative women in the first screening round in 2012–2015, 33.3% (8/24) did not attend the second screening round in 2017–2020. Of the originally hrHPV-positive women in 2012–2015, 38.1% (24/63) did not attend the next screening round in 2017–2020 or left a follow-up during this screening round unattended. Of these patients, 8 (12.7%) had a negative hrHPV test result at the last follow-up they attended but 16 (25.4%) were still hrHPV positive when they dropped out of the screening protocol (data not shown).

## 4. Discussion

The results of the present study are in agreement with the previously published series in the sense that no endocervical glandular malignancies were found among patients with endocervical cell atypia and a negative hrHPV result, reflecting that the majority of the endocervical adenocarcinomas are associated with hrHPV infection [8,9,10]. In our study, 0.13% of the pap tests were reported as endocervical glandular atypia, which is in the lower range compared to the previously published incidence rates of between 0.1% and 1.84% [21,22,23,24]. This variation in reported incidence is not surprising, since the interobserver agreement between pathologists, especially regarding mild cytological changes, is known to be relatively poor, and differentiating between cytological glandular features and squamous features has also proved to be challenging [25,26]. In the present study, 37.3% of the cases diagnosed with mild (not otherwise specified) endocervical glandular atypia were hrHPV negative, which is significantly less than the 79.8% reported by Chen et al. [23].

In addition to the lack of glandular malignancies, in the present study, there were no histological HSIL or SCC among the cases with endocervical glandular atypia and a negative hrHPV result. This is supporting the previous findings, according to which over 60% of the mild cytological glandular atypias with or without mild squamous atypia represent benign changes, or low-grade squamous histological lesions [22,27,28,29].

In our study, behind the cytological endocervical glandular diagnoses, with or without combined squamous cytological atypia, 68.4% of the clinically significant lesions (HSIL, AIS or worse) were purely of squamous origin. This might be caused by the extension of HSIL to endocervical glands, which is known to be a common cause of false cytological glandular diagnoses, or simply the result of the everyday struggle pathologists have in separating the cytological glandular features from squamous features [25,26,30]. Nevertheless, the amount of squamous lesions behind cytological endocervical diagnoses in our study, was roughly in the same range as the 73% and 77% previously reported [24,31].

In previous studies, the hrHPV test has been more often positive for cancer and precancerous lesions than cytology, and the difference in performance has been more pronounced among cases subsequently diagnosed with AIS or EAC than CIN3 or SCC [11,12,15,16]. Additionally, the implementation of hrHPV testing has led to an earlier detection of CIN2 lesions or worse, and reduced the detection of CIN3 lesions and cervical cancer during follow-ups in comparison to cytology alone [13,14,31,32,33]. In the ARTISTIC study, however, the routine HPV testing did not significantly improve the recognition of CIN3 lesions compared to liquid-based cytology [34]. Nevertheless, a negative hrHPV test result during screening was shown to predict a negative outcome better than the cytology [11,13], which was also conversely seen in our study, since there were no high-grade histological lesions among hrHPV negative samples with cytological atypia [13,33].

Similarly, in the present study, only two malignancies were found during the second screening rounds. Both lesions represented AIS, and their histological diagnoses were not reached until 5 years and 9 months and 6 years and 11 months after the initial hrHPV positivity and cytological glandular atypia at the study baseline. 

Although the participation rate for screening (70.8%) in the present study was similar to that previously reported in Finland [2], the high drop-out rate in the study was surprising, and possibly affected the end results. Of the originally hrHPV-positive patients, 25.4% dropped out of the screening protocol with a positive hrHPV test result at their last follow-up. This might have led to some cancers or precancerous lesions left undiagnosed. There was one death due to other reasons. Since different regional and private practice databases in Finland do not communicate, we do not know if the other patients dropping out the screening protocol have moved, were treated elsewhere or if they simply chose not to participate.

Our database search revealed one hrHPV-negative mucinous, gastric type adenocarcinoma case missed by the primary HPV screening during the study years 2014–2021. Since the hrHPV test at the screening was negative, no follow-ups were scheduled for the patient. The cancer was later diagnosed on a gynecological check-up for other reasons.

Since, in previous studies, 22% of the EAC and around 15% of the AIS were negative both in cytology and in hrHPV test, it can be only speculated if including the cytological sample to the screening protocol would have led to an earlier diagnosis in this case [11,12]. It has been calculated that adding a cytological sample to hrHPV screening would lead to an earlier detection of, at most, five cases per million women in a year [12]. Adding a p16/Ki67 dual-stain to screening has been reported to increase the detection rate of histological HSIL among hrHPV positive and cytology negative cases [35]. A positive Hepika test on a cytological sample seems to have a high sensitivity for invasive carcinoma, both squamous and glandular, in comparison to precursor lesions [36]. Since p16 and Hepika tests are both surrogate markers for hrHPV infection, adding them to the screening protocol in selected cases would probably increase the rate of, or at least provide an earlier detection for endocervical adenocarcinomas. Since the gastric type endocervical adenocarcinomas are practically always hrHPV negative, these tests are not likely to be of benefit, in their diagnosis [9,10].

In our series, all AIS and EAC were positive with either HPV16 or HPV18. Out of the HSIL, 42.9% were positive with an hrHPV type other than HPV16 or HPV18. In the statistical analyses, the HPV types (16, 18 or other) or combinations of them did not provide a prediction specifically relating to endocervical malignancies or to squamous lesions. This may have been due to the small number of positive cases, which may also have been the reason why the positive cases were not analysed further.

## 5. Conclusions 

In conclusion, in this study of two consecutive HPV-primary screening rounds, among 87 women diagnosed with AEC, NOS or AEC, FN in cytology, three EAC, two AIS, one AIS + HSIL and 13 HSIL in were found in histology. All the EAC and AIS were either HPV16 or HPV 18 positive. No high-grade histological lesions were found among the hrHPV-negative patients diagnosed with cytological endocervical cell atypia. A later database search revealed one HPV-negative gastric-type mucinous adenocarcinoma that was missed by the primary HPV screening.

## Figures and Tables

**Table 1:** The high-grade histological lesions detected on the two screening rounds during 2012–2015 and 2017–2020, including the hrHPV genotypes and the initial cytological diagnoses.

HPV Genotype ^1^	Cytological Diagnosis ^2^	Histological Lesion ^3^
other	AEC, NOS	HSIL
other	AEC, NOS	HSIL
other	AEC, NOS	HSIL
other	AEC, NOS	HSIL
16	AEC, NOS	AIS
16	AEC, NOS	HSIL
16, 18, other	AEC, NOS	HSIL, later AIS
other	AEC, NOS + ASC-US	HSIL
16, other	AEC, NOS + ASC, US	HSIL
16	AEC, NOS + ASC-H	HSIL
16, 18	AEC, NOS + ASC-H	HSIL
16, other	AEC, NOS +HSIL	HSIL
16	AEC, NOS + HSIL	HSIL
16	AEC, NOS +HSIL	EAC + LSIL
18	AEC, NOS + HSIL	EAC + LSIL
16	AEC, FN	HSIL
other	AEC, FN	HSIL
16	AEC, FN	EAC
18	AEC, FN	AIS

^1^ In the screening hrHPV genotypes 16 and 18 were reported separately and the rest of the 14 genotypes were recognized by The Abbot RealTime hrHPV PCR assay as “other hrHPV than HPV16 or HPV18”. ^2^ AEC, NOS (Atypical Endocervical Cells, NOS), AEC, FN (Atypical Endocervical Cells, Favor Neoplastic), AIS (Adenocarcinoma in Situ), EAC (Endocervical Adenocarcinoma), ASC-US (Atypical Squamous Cells, Undetermined Significance), LSIL (Low-Grade Squamous Intraepithelial Lesion), HSIL (High-Grade Squamous Intraepithelial Lesion), ASC-H (Atypical Squamous Cells, Cannot Exclude an HSIL). ^3^ AIS (Adenocarcinoma in Situ), EAC (Endocervical Adenocarcinoma), LSIL (Low-Grade Squamous Intraepithelial Lesion) including Cervical Intraepithelial Lesion (CIN) 1 and condyloma, HSIL (High-Grade Squamous Intraepithelial Lesion) including CIN2 and CIN3.

**Table 2:** Cytological diagnoses with HPV status and the follow-up data on two cervical cancer screening rounds (2012–2015 and 2017–2020) among patients with cytological endocervical glandular diagnosis at the first screening round.

	AEC, NOS +/− ASC-US/LSIL *	AEC, NOS +/− ASC-H/HSIL **	AEC, FN *** +/− ASC-US/LSIL	TOTAL
	HPV+/HPV− (*n* = 44/*n* = 25)	HPV+/HPV− (*n* = 9/*n* = 0)	HPV+/HPV− (*n* = 9/*n* = 0)	HPV+/HPV− (*n* = 62/*n* = 25)
ATTENDANCE				
Not attending a follow-up during the 1st screening round	9/7	0/NA ****	2/NA	11/7
Not attending a follow-up during the 2nd screening round	18/8	2/NA	4/NA	24/8
HPV negative on a follow-up during the 1st screening round	8/15	0/NA	0/NA	8/15
HPV negative on a follow-up during the 2nd screening round	16/15	5/NA	2/NA	23/15
FINAL HISTOLOGY				
Adenocarcinoma (EAC)	0/0	0/NA	1/NA	1/0
Adenocarcinoma in situ (AIS)	1/0	0/NA	1/NA	2/0
High-grade intraepithelial lesion (HSIL)	7/0	4/NA	2/NA	13/0
Low-grade intraepithelial lesion (LSIL)	9/0	4/NA	1/NA	13/1
EAC + LSIL	0/0	2/NA	0/NA	2/0
AIS + HSIL	1/0	0/NA	0/NA	1/0
Benign histology	12/2	0/NA	5/NA	17/2
Follow-up coming	3/1	0 NA	0/NA	3/1

* AEC, NOS (Atypical Endocervical Cells, NOS), ASC-US (Atypical Squamous Cells, Undetermined Significance), LSIL (Low-Grade Intraepithelial Lesion) including condyloma and Cervical Intraepithelial Lesion 1 (CIN1). ** ASC-H (Atypical Squamous Cells, Cannot Exclude an HSIL), HSIL (High-Grade Intraepithelial Lesion. *** AEC, FN (Atypical Endocervical Cells, Favor Neoplastic). **** NA (not applicable). There were no combination diagnoses of AEC, FN + ASC-H/HSIL.

## Data Availability

The data that support the findings of this study are available from the corresponding author upon reasonable request.
